# High expression of *MAP7* predicts adverse prognosis in young patients with cytogenetically normal acute myeloid leukemia

**DOI:** 10.1038/srep34546

**Published:** 2016-09-30

**Authors:** Lin Fu, Huaping Fu, Lei Zhou, Keman Xu, Yifan Pang, Kai Hu, Jing Wang, Lei Tian, Yuanyuan Liu, Jijun Wang, Hongmei Jing, Wenrong Huang, Xiaoyan Ke, Jinlong Shi

**Affiliations:** 1 Department of Hematology and Lymphoma Research Center, Peking University, Third Hospital, Beijing, 100191, China; 2 Department of Biomedical Engineering, Chinese PLA General Hospital, Beijing, 100853, China; 3 Blood Diseases Institute, Xuzhou Medical University, Xuzhou, 221002, China; 4 Department of Nuclear Medicine, Chinese PLA General Hospital, Beijing, 100853, China; 5 Department of Hematology, Chinese PLA General Hospital, Beijing, 100853, China; 6 College of Medical Laboratory Science and Technology, Harbin Medical University, Daqing, 163319, China; 7 Department of Medicine, William Beaumont Hospital, Royal Oak, MI, 48073, USA

## Abstract

Microtubule-associated protein 7 (*MAP7*) plays an important role in cancer cells. In this study, we identified the prognostic significance of *MAP7* expression in cytogenetically normal acute myeloid leukemia (CN-AML) patients (aged <60 years) based on several microarray datasets. In the first group (n = 129), high *MAP7* expression (*MAP7*^high^) was associated with adverse overall survival (OS; *P* = 0.0441) and event-free survival (EFS; *P* = 0.0114) compared with low *MAP7* expression (*MAP7*^low^). In addition, the prognostic significance of *MAP7* was confirmed by European Leukemia Net (ELN) intermediate-I genetic categories and multivariable analysis. In the second independent group of CN-AML patients (aged <60 years), *MAP7*^high^ was also associated with adverse OS (n = 88, OS; *P* = 0.00811). To understand the inherent mechanisms of *MAP7*’s prognosis, we investigated genome-wide gene/microRNA expression signatures associated with *MAP7* expression. Several known oncogenic genes/microRNAs and anti-oncogenic genes/microRNAs were disordered in *MAP7*^high^ CN-AML patients. In conclusion, *MAP7*^high^ is an adverse prognostic biomarker for CN-AML, which may be attributed to the distinctive genome-wide gene/microRNA expression and related cell signaling pathways.

Cytogenetically normal acute myeloid leukemia is the largest cytogenetic subset in AML patients^1^. CN-AML is defined by the lack of detectable chromosome abnormalities and other sensitive prognostic biomarkers of chromosome abnormalities. However, they may contain insidious mutations, aberrantly expressed genes, or microRNAs that are potential prognosticators. Studies have already indicated several factors associated with favorable or poor outcomes. The former group includes *NPM1* and double *CEBPA* mutations. The latter group includes *FLT3*-ITD and high expression of *ERG*^2^, *BAALC*^2^, *WT1, DNMT3B*^3^*, ITPR2*^4^*, MAPKBP1*^5^, and *ATP1B1*^6^, as well as low expression of *LEF1*^7^. Such biomarkers can be useful indicators for risk stratification as well as provide insight into the pathogenesis of CN-AML and thus inspire novel targeted therapies.

In this study, we used multiple types of Gene Expression Omnibus (GEO) microarray datasets and different bioinformatic approaches to systematically screen for possible prognostic biomarkers. Gene signatures with both aberrant expression and significant prognostic values were filtered out (5 genes), including *MAP7*. Overlapping our results with previous reports included in a 24-gene AML prognostic signature^8^, *MAP7* was the only gene that was identified in common across all reports (Supplementary Figure 1).

*MAP7* is one of the microtubule-associated proteins (*MAPs*) and is predominantly expressed in cells of epithelial origin. *MAPs* are involved in microtubule dynamics and are indispensable in cell division, motility, differentiation and other important cellular and intracellular activities^9^. *MAP7* has shown important prognostic value in several types of malignancy. Recently, *MAP7*^high^ was shown to be associated with tumor recurrence and poor prognosis in stage II colon cancer patients^10^. However, the possible impact of *MAP7* expression on the prognosis of CN-AML has yet to be examined. Therefore, we aimed to explore the prognostic significance and mechanism of action for *MAP7* in CN-AML.

## Results

### Expression of MAP7 in AML patients and normal controls

**Figure 1:**
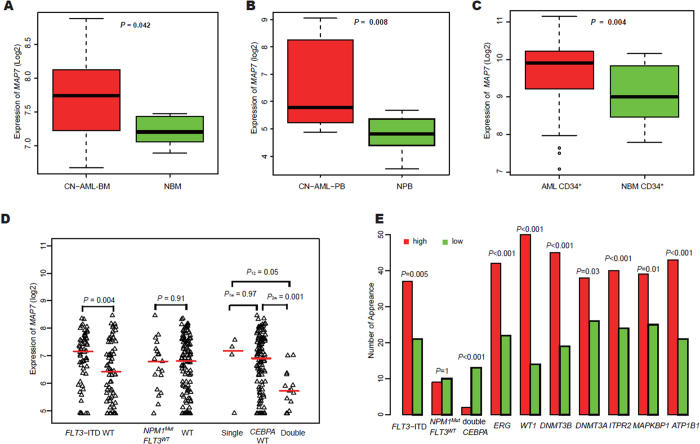
Differential expression of *MAP7*. (**A**) CN-AML-BM cases (n = 116) compared with NBM samples (n = 5); (**B**) CN-AML-PB cases (n = 7) compared with NPB samples (n = 10); (**C**) AML CD34^+^ cells (n = 46) compared with NBM CD34^+^ cells (n = 31); (**D**) Expression of *MAP7* in CN-AML patients with *FLT3*-ITD and the mutation of *NPM1* and *CEBPA*; (**E**) Associations between *MAP7* expression and known prognostic biomarkers in CN-AML patients.

A microarray dataset of BM samples was used for expression analysis, including 116 CN-AML BM and 5 normal bone marrow (NBM) samples (GEO accession number *GSE1159*). Remarkably higher expression of *MAP7* was evident in CN-AML BM than NBM (*P* = 0.042) (Fig. 1A). Overexpression of *MAP7* was further validated by another microarray dataset of peripheral blood (PB) samples [7 CN-AML PB samples (PB samples contained 70–97% blast cells) *vs.* 10 normal PB (NPB) samples (*P* = 0.008), GEO accession number *GSE9476*] (Fig. 1B). Moreover, a third database that included AML CD34^+^ cells (n = 46) and NBM CD34^+^ cells (n = 31) derived from potential donors for allogeneic BM transplantation was used for the *MAP7* expression analysis (GEO accession number *GSE30029*). The results showed a significant increase in expression of *MAP7* in AML CD34^+^ cells compared with NBM CD34^+^ cells (*P* = 0.004) (Fig. 1C). Furthermore, *MAP7*^high^ was found in CN-AML (n = 116) among the different subgroups of AML patients as follows: 19 CBFβ-MYH11 patients, 10 patients with complex karyotypes, 17 patients with MLL-translocation, 86 patients with other findings, 18 PML-RARA patients, and 22 AML-ETO patients. Additionally, *MAP7*^high^ was found in 5 NBM patients (Supplementary Figure 2). These findings showed that *MAP7* was widely expressed at high levels in CN-AML patients.

### Associations between MAP7 expression and other prognostic biomarkers in CN-AML

The 129 CN-AML patients were further divided into subgroups by the presence of *FLT3*-ITD and mutation status of *NPM1* and *CEBPA*. Levels of *MAP7* expression were compared among different subgroups (GEO accession number *GSE6891*) (Fig. 1C). *MAP7* was significantly more highly expressed in samples with *FLT3*-ITD and single and wild-type *CEBPA* compared with samples without *FLT3-*ITD and samples with double *CEBPA* mutations (*P* = 0.004, *P* = 0.05, and *P* = 0.001, respectively, Fig. 1D). No significant differences were detected between *NPM1*-mutated (no *FLT3*) and wild-type samples (*P* = 0.91) or between single *CEBPA*-mutated and wild-type samples (*P* = 0.97, Fig. 1E).

### Differences in gene mutation and expression profiles between MAP7^high^ and MAP7^low^ groups

In the 129-patient cohort, patients in the *MAP7*^high^ group were more likely to carry an *FLT3*-ITD mutation and less likely to carry a double *CEBPA* mutation (*P* = 0.005, *P* = 0.005) compared with the *MAP7*^low^ group. No additional links between *MAP7* expression and other mutations were found. *MAP7*^high^ patients with CN-AML were more likely to have a higher expression of *ERG, WT1, DNMT3B, DNMT3A, MAPKBP1, ITPR2* and *ATP1B1* mutations than *MAP7*^low^ patients (*P* = 0.0004, *P* < 0.0001, *P* < 0.0001, *P* = 0.03, *P* = 0.01, *P* = 0.005, and *P* < 0.001, respectively). See Table 1 and Fig. 1D.

### MAP7^high^ was associated with adverse outcomes

**Figure 2:**
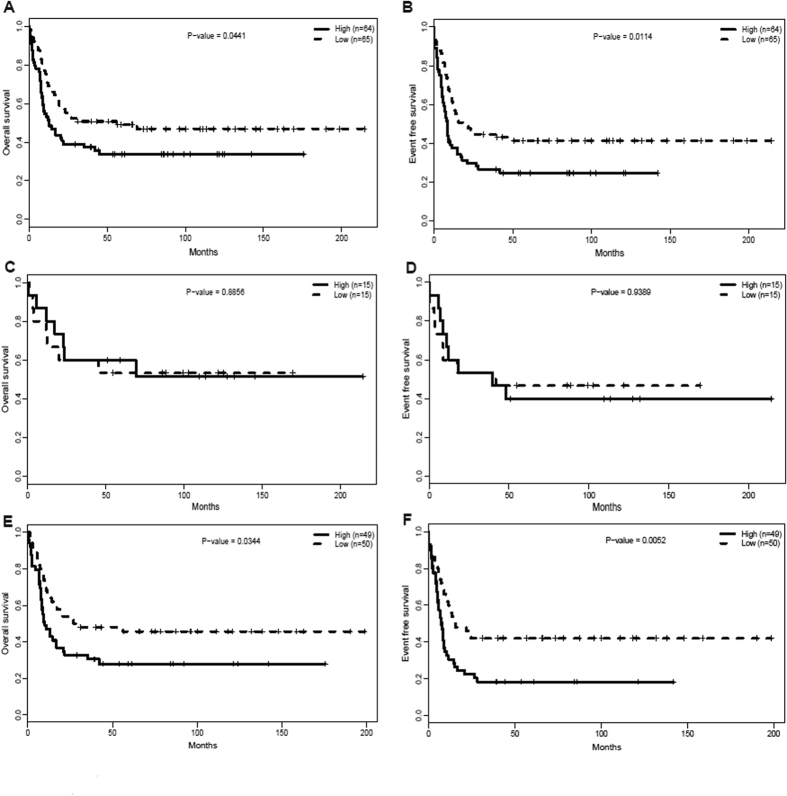
Prognostic value of *MAP7* expression. (**A**) OS and (**B**) EFS in the 129 CN-AML patients; (**C**) OS and (**D**) EFS in the ELN Favorable category; (**E**) OS and (**F**) EFS in the ELN Intermediate-I category.

In the 129-CN-AML patient cohort, the *MAP7*^high^ group had markedly shorter OS (Fig. 2A, *P* = 0.0441) and EFS (Fig. 2B, *P* = 0.0114) compared with the *MAP7*^low^ group. The associations between *MAP7* expression and prognostic significance within the European Leukemia Net (ELN) favorable group and intermediate-I genetic group were also separately analyzed. Within the ELN favorable group (n = 30), no significant difference was observed in OS (Fig. 2C, *P* = 0.8856) or EFS (Fig. 2D, *P* = 0.9389) between the *MAP7*^high^ and *MAP7*^low^ patients. In the ELN Intermediate-I group (n = 99), however, *MAP7*^high^ patients had a significantly shorter OS (Fig. 2E, *P* = 0.0344) and EFS (Fig. 2F, *P* = 0.0052) than *MAP7*^low^ patients.

### MAP7 expression was associated with shorter OS and EFS in multivariable analyses

After adjusting for the impact of known risk factors, we performed multivariable analyses to confirm the prognostic significance of *MAP7* expression. In the multivariable models for OS and EFS, *MAP7*^high^ had adverse impacts on OS (*P* = 0.03, Table 2) as well as EFS (*P* = 0.01, Table 2). The other factors negatively correlated with EFS were *NPM1* wild-type and *FLT3*-ITD mutations (*P* = 0.01 and *P* = 0.04, respectively, Table 2).

### MAP7^high^ was associated with adverse outcomes in the second independent CN-AML group

We studied an independent group of 88 previously untreated CN-AML patients (aged <60 years). Patients with the FAB-M5 mutation were found to have more *MAP7*^low^ among all FAB subtypes (*P* = 0.033). In this cohort, *MAP7*^high^ patients with CN-AML were more likely to have higher expressions of *ERG, WT1, DNMT3B, DNMT3A, ITPR2, MAPKBP1* and *ATP1B1 (P* < 0.001, *P* < 0.001, *P* < 0.001, *P* = 0.02, *P* < 0.001, *P* < 0.001, and *P* < 0.001, respectively) and lower expression of *LEF1 (P* = 0.001) compared with *MAP7*^low^ patients (Supplementary Table 1). In addition, *MAP7*^high^ patients had significantly shorter OS rates than *MAP7*^low^ patients (n = 44 vs. n = 44, *P* = 0.00811; Supplementary Figure 3).

### Associations between genome-wide gene-expression profiles and MAP7 expression

**Figure 3:**
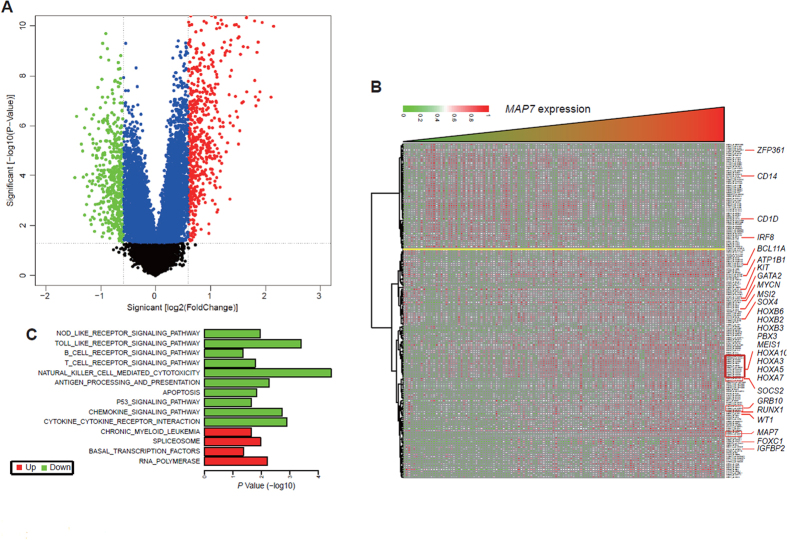
Genome-wide gene expression profile and cell signaling pathways associated with *MAP7* expression is shown. (**A**) Volcano plot of differential gene expression; *MAP7*^high^ and *MAP7*^low^ were marked by red and green circles, respectively; (**B**) Expression heat map of associated genes; (**C**) Cell signaling pathways.

To further assess the role of *MAP7* in CN-AML, we derived *MAP7*-associated gene expression profiles by microarray analysis. We first identified 586 up-regulated and 482 down-regulated genes that were significantly associated with *MAP7* expression (*P* < 0.05, Fold Change = 1.5, Fig. 3A). With a more rigorous analysis (Fold Change = 2, and profiles with NA values were all deleted), 180 genes were filtered and presented in an aberrant expression heat map (Fig. 3B). The up-regulated genes included the following: 1) genes involved in leukemogenesis (*MYCN, Sox4*^11^), tumorigenesis promoters (*HOXA2, 3, 5, 7, 10* and *HOXB2, 3, 6*^12^*,BCL11A*^13^, *FOXC1*^14^, *RUNX1*^15^, *Pbx3*^16^ and *Meis1*^16^), and tyrosine kinase genes (*c-KIT, GRB10*); 2) independent adverse prognostic factors in AML including *WT1, RUNX1*^17^*, SOCS2*^18^*, GATA2*^19^*, ATP1B1*^6^ and *MSI2*^20^; and 3) genes correlating with chemotherapy resistance in adult AML patients (*IGFBP2*^21^). The down-regulated genes included the following: 1) immune system activators such as *CD14* and *CD1d*; 2) hematopoietic tumor suppressor *IRF8*^22^; and 3) *ZFP36L1*, a promotor of monocyte/macrophage differentiation that represses *CDK6*^23^. These dysregulated genes might explain the correlation between *MAP7* and the prognosis of CN-AML.

Tumorigenesis is closely associated with different cell signaling pathways, each composed of many genes. To assess the biological features associated with *MAP7*, cell signaling pathways in *MSigDB* were evaluated, and the mean expression of all genes in a pathway was used to quantize its expression level (Fig. 3C). Cell signaling pathways involved in “RNA polymerase”, “basal transcription factors”, “spliceosome”, and “chronic myeloid leukemia” were significantly up-regulated, whereas immune response pathways such as “cytokine-cytokine receptor interaction”, “chemokine signaling”, “antigen processing and presentation”, “natural killer-cell-mediated cytotoxicity”, “T-cell receptor signaling”, “B-cell receptor signaling”, “toll-like receptor signaling”, and “NOD-like receptor signaling” were down-regulated. Apoptotic pathways including “p53 signaling” and “apoptosis” were also down-regulated. These findings were consistent with the dysregulated gene expressions, collectively, these genes and pathways might be involved in the development of CN-AML.

### Associations between genome-wide microRNA profiles and MAP7 expression

**Figure 4:**
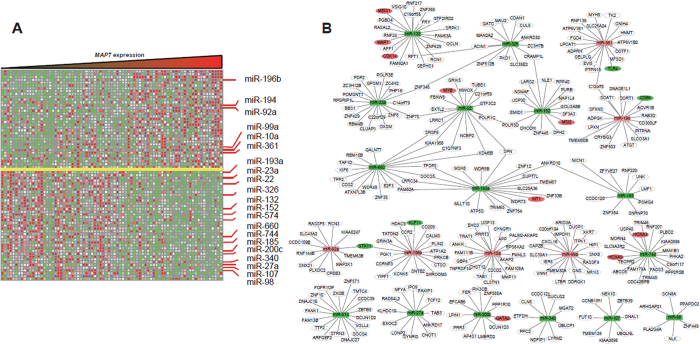
Genome-wide microRNA expression profile associated with *MAP7* expression is shown. (**A**) Expression heat map of associated microRNAs; (**B**) Candidate miRNAs with their target genes.

An analysis of *microRNA* genome-wide profiles revealed 145 microRNAs that were strongly associated with *MAP7* expression (*P* < 0.05) (Fig. 4A). First, *MAP7*^high^ was positively correlated with levels of *miR-196b, miR-92a, miR-99a, miR-10a, miR-361* and *miR-194*. In previous reports, these microRNAs were shown to have important tumor-promoting properties. *miR-196b* targets tumor-suppressor genes such as *Fas*^24^, and overexpression of *miR-196b* is associated with aggressive **leukemia in mice and a poor prognosis in AML.** *miR-92a* promotes cell proliferation in acute promyelocytic leukemia and induces erythroleukemia through *p53* down-regulation^25^. *miR-99a* serves as a potential oncogene in pediatric myeloid leukemia^26^. Overexpression of *miR-10a* is associated with poor OS in AML patients^27^. The level of *miR-361* was found to be decreased after chemotherapy^28^. An increased expression of *miR-194* is associated with an increased risk of a poor prognosis in CN-AML patients^29^. Second, *miR-193a, miR-23a, miR-22, miR-326, miR-132, miR-152, miR-574, miR-660, miR-744, miR-185, miR-200c, miR-340, miR-27a, miR-107* and *miR-98* were down-regulated. In our previous study, we showed that *miR-193a* targeted *c-kit*^30^. The down-regulation of *miR-193a* could lead to a higher expression of *c-kit*, consistent with the above-stated gene-expression profiles. Other microRNAs in this group have important tumor-suppression roles. *miR-23a* targets the *BCR/ABL* oncogene in CML^31^. *miR-22* regulates the expression of oncogenic *NET1* in CML^32^. *miR-326* represses the oncogenic Hedgehog pathway in CML by targeting the signal transducer *Smo*^33^. *miR-132* targets the *p53*-down-regulator *SIRT1*, which in turn promotes *p53* expression^34^*. miR-152* is crucial for the anti-tumor effect of natural killer cells by up-regulating *HLA-G*^35^. *miR-574* is a tumor suppressor in imatinib-resistant CML^36^. *miR-660* is down-regulated in lung cancer patients, and it inhibits lung tumorigenesis by targeting the *MDM2-p53* interaction^37^. Proto-oncogene *eEF1A2* is a target of *miR-744*^38^. *miR-185* suppresses tumor proliferation in breast cancer by directly targeting *E2F6* and *DNMT1* and indirectly up-regulating *BRCA1*^39^. *miR-200c* inhibits breast cancer proliferation by targeting *KRAS*^40^. *miR-340* inhibits glioblastoma cell proliferation by suppressing *CDK6, cyclin-D1* and *cyclin-D2*^41^. *miR-27a* functions as a tumor suppressor in acute leukemia by regulating *14-3-3θ*^42^. Epigenetic silencing of *miR-107* regulates *CDK6* expression in pancreatic cancer^43^. *miR-98* inhibits tumor angiogenesis and invasion by targeting *ALK4* and *MMP11*^44^.

To explore the dynamic *microRNA-mRNA* regulatory relationship associated with *MAP7* expression in CN-AML, we used microRNA-target prediction algorithms to analyze the target genes of the microRNAs mentioned above^45^ (Fig. 4B). In the group of up-regulated microRNAs, *TLR4, CD86* and *KLF11* were the target genes of *miR-361, miR-194* and *miR-196b,* respectively. *STX11* was a common target of *miR-92a* and *miR-196b.* These target genes have been proven to exert important anti-tumor effects as follows: *TLR4* and *CD86* can activate the anti-tumor effect of NK-cells, *KLF11* is a tumor suppressor gene in myelodysplastic syndrome (MDS)^46^, and *STX11* defects may be associated with MDS and AML^47^. In the group of down-regulated microRNAs, *MEIS1, MAP7* and *CDK14* were the target genes of *miR-132. MSI2* and *MYB* were the target genes of *miR-152* and *miR-22,* respectively. *WT1* was the target gene of *miR-193a. HOXA3* and *HOXA9* were the target genes of *miR-744. GATA2* was the target gene of *miR-200c*. Collectively, alterations of the microRNA profiles might contribute to the prognosis of *MAP7* through their regulation of target genes.

## Discussion

CN-AMLs constitute the largest proportion of all primary AMLs. The leukemic blasts of CN-AML patients contain none of the detectable chromosome abnormalities that are traditionally sensitive prognosticators. Identification of universal prognostic biomarkers has been a key focus in CN-AML research. *MAP7*, a gene involved in microtubule stabilization and epithelial cell differentiation, had shown promising prognostic values in our serial experiments. First, we found that *MAP7* expression was up-regulated in two independent CN-AML cohorts. This finding indicated that *MAP7* might play an active role in leukemogenesis. In the first cohort of 129 patients (aged <60 years), our study demonstrated that *MAP7*^high^ was associated with shorter OS and EFS. In this cohort, we found that *MAP7*^high^ was associated with the presence of other adverse prognosticators, such as *FLT3*-ITD, and high *ERG, WT1, DNMT3B, ITPR2, MAPKBP1* and *ATP1B1* expression. After adjusting for known prognosticators by multivariable analyses, the association of *MAP7*^high^ with adverse OS and EFS still existed. These results indicated that *MAP7*^high^ might be an adverse prognostic biomarker and could substitute other adverse factors. Using ELN genetic categories, our results suggested that the prognostic impact of *MAP7* expression was most pronounced in the ELN intermediate-I genetic group, and *MAP7* expression could therefore be used to refine the risk stratification for these patients.

Given that the first CN-AML group (n = 129, aged <60 years) included a small number of patients who had received Allogeneic Hematopoietic Stem Cell Transplantation (AlloSCT), the prognostic value of *MAP7* was further confirmed in the second independent group of CN-AML patients without AlloSCT who received intensive double induction and consolidation chemotherapy (n = 88, aged <60 years). Thus, it seemed that *MAP7*^high^ could be used as an adverse prognostic biomarker for CN-AML patients.

CN-AML patients show a degree of genetic uniformity, which facilitates the identification of new biomarkers but limits the scope of their application. However, *MAP7* was the only gene that was identified by overlapping our results with previous reports included in a 24-gene AML prognostic signature^8^, indicating that *MAP7*^high^ may be an adverse biomarker in both CN-AML and AML.

Genome-wide expression profile analysis showed that in the *MAP7*^high^ CN-AML patients, genes related to cell proliferation regulation were up-regulated, in particular, *c-KIT* and *MYCN*. Genes that were independent adverse prognostic factors were also overexpressed. By contrast, genes related to tumor suppression and immune activation were down-regulated in *MAP7*^high^ patients. Several important cell signaling pathways that promote cell proliferation or contribute to leukemogenesis, such as “RNA polymerase”, “basal transcription factors” and “chronic myeloid leukemia” were up-regulated, whereas some immune activation signaling pathways, such as “toll-like receptor signaling” and “NOD-like receptor signaling” were down-regulated. “*P53* signaling” and “apoptosis” pathways were also down-regulated. The down-regulation of these pathways might explain the immune escape and apoptosis blockage in *MAP7*^high^ CN-AML patients.

Recently, it has been found that microRNAs play an important role in regulating the lineage differentiation of hematopoietic cells. MicroRNAs modulate the expression of oncogenes or tumor suppressors. In our study, the *MAP7*-associated microRNA profile revealed that in *MAP7*^high^ CN-AML patients, some oncogenic microRNAs such as *miR-196b* and *miR-99a* were up-regulated, whereas anti-tumor microRNAs such as *miR-193a* and *miR-27a* were down-regulated.

These dysregulated genes and/or microRNAs could potentially interact, contributing to leukemogenesis. For example, among the dysregulated genes, *FOXC1*, a gene almost exclusively associated with expression of the *HOXA/B* locus, could block monocyte/macrophage differentiation and enhance clonogenecity^14^. Another dangerous liaison observed among *Pbx3, Meis1* and *Hoxa9* contributed to leukemogenesis^16^. In the network of dysregulated microRNA-mRNA/pathways, the following associations were made: 1) *miR-132* could target *MEIS1* and *MAP7*; 2) *miR-193a* could target *WT1* and *c-kit*; 3) *miR-744* could target *HOXA3*; 4) *miR-152* could target *MSI2*; 5) *miR-200c* could target *GATA2*; 6) *miR-92a* and *miR-660* could cause *p53* down-regulation directly or indirectly in AML and lung cancer; and 7) *miR-132* could promote *p53* down-regulation by targeting *SIRT1.* These microRNA expression profiles were consistent with the observed gene-expression profiles in the network of dysregulated-mRNA/pathways. The above findings of *MAP7*-associated gene/microRNA profiles and cell signaling pathways helped explain the leukemogenesis process and the adverse outcomes in *MAP7*^*high*^ patients with CN-AML.

With respect to recent findings that promoter methylation of certain genes played an important role in tumorigenesis^48^ and *MAP7* expression and was significantly associated with the increased expression of *DNMT3A* and *DNMT3B* in both cohorts, we explored the association between *MAP7* expression levels and the methylation levels of genome-wide gene promoters. Although genome-wide deregulated gene promoter methylation profiles were found in CN-AML patients when high *vs* low *MAP7* expressers were compared, none of the genes that contributed to leukemogenesis in AML were found (data not shown). Overall, these data suggest that although *MAP7*^*high*^ is a potentially predictive marker in CN-AML, genome-wide deregulated gene promoter methylation profiles do not provide insights into the pathogenesis of CN-AML.

In summary, our study is the first to provide evidence that *MAP7*^high^ is associated with adverse outcomes in CN-AML patients, even after adjusting for other known molecular risk factors. Previous findings demonstrated the consistency and validity of microarray expression data from quantitative real-time PCR (qPCR). For example, in the second cohort, the microarray expression data and the qPCR data of *LEF1* expression were in good agreement. Compared with NBM, *MAP7* is widely expressed at a higher level in CN-AML patients, and its expression can be more readily measured in clinical settings. Its overexpression may be a valuable new marker for risk stratification of CN-AML patients. Moreover, distinctive gene/microRNA expression patterns in CN-AML patients provide insights into the pathogenesis processes associated with varying *MAP7* expression levels. Our results also indicated that *MAP7* may be a promising therapeutic target for CN-AML.

## Methods

### Patients and treatment

The first group included 129 untreated primary CN-AML patients diagnosed between 1990 and 2008 (median age, 46 years; range, 16–59 years). Per diagnosis, all were uniformly treated based on study protocols of the Dutch-Belgian Cooperative Trial Group for Hematology Oncology (HOVON) (details of the therapeutic protocol available at http://www.hovon.nl). Normal karyotype was established with a conventional cytogenetic examination of at least 20 metaphases from bone marrow (BM). All samples contained 80–100% blast cells after thawing. *NPM1, CEBPA, IDH1*, and *IDH2* mutations, *FLT3*-ITD and tyrosine kinase domain mutations (*FLT3*-TKD [D835]) were examined by RT-PCR assays. All clinical, cytogenetic, molecular information, as well as gene expression profiles of the 129 patients, can be publicly downloaded (www.ncbi.nlm.nih.gov/geo, accession number *GSE6891*). This research was approved by the institutional review boards at Weill Cornell Medical College and Erasmus University Medical Center, and written donor informed consent was obtained in accordance with the Declaration of Helsinki. The methods were carried out in accordance with the approved guidelines.

The second independent group of 88 CN-AML patients (median age: 47 years; range: 17–59 years) also received uniform treatment provided by the multicenter AMLCG-1999 trial. These patients received intensive double induction and consolidation chemotherapy. Gene expression data are publicly available (http://www.ncbi.nlm.nih.gov/geo/, accession number *GSE12417*). The AMLCG-1999 clinical trials were approved by the local institutional review boards, and informed consent from all patients was obtained in accordance with the Declaration of Helsinki. The methods were carried out in accordance with the approved guidelines.

### Microarray analyses

Gene/microRNA expression and methylation data were previously published (accession number *GSE1159,GSE9476, GSE30029, GSE6891* and *GSE12417* for gene expression). The Cancer Genome Atlas (TCGA) database was used for mRNA/microRNA expression and genome-wide promoter methylation. Briefly, gene expression data were obtained by Affymetrix Human Genome 133 plus 2.0 and U133A Gene Chips. All designs and quality control of the microarray experiment and data normalization were in line with the standard Affymetrix protocols. RNA-Seq data of mRNA/microRNA and genome-wide promoter methylation levels were derived from TCGA obtained by whole-genome high-throughput sequencing and Illumina 450 K chips, respectively, which provided 73 CN-AML patients with all data for mRNA, microRNA and methylation. Patients with *MAP7* expression values (whether microarray in *GEO* or RNA-Seq in TCGA) above the median for all patients were classified as having *MAP7*^high^, and the others were considered to have *MAP7*^low^. *ERG, BAALC, LEF1, WT1, DNMT3A, DNMT3B, MAPKBP1, ITPR2,* and *ATP1B1* expression levels were also determined from the microarray data.

### Statistical analyses

OS was defined as the time from date of diagnosis to death due to any causes. EFS was defined as the time from date of diagnosis to removal from the study because of the absence of complete remission, relapse or death. First, because *MAP7* expression was found to be normally distributed in 129 CN-AML patients (Supplementary Figure 4A), we subdivided 129 CN-AML patients into four quartiles (Q1: <25%, Q2: 25~50%, Q3: 50~75%, Q4: >75%) based on *MAP7* expression values to determine the best classification method for this group. Second, we found that no significant difference was observed between Q1 and Q2 (OS: Q12, *P* = 0.852; EFS: Q12, *P* = 0.777), and the same result was also observed between Q3 and Q4 (OS: Q34, *P* = 0.342; EFS: Q34, *P* = 0.524). Although this result was also observed between Q2 and Q3 (OS: Q23, *P* = 0.283; EFS: Q23, *P* = 0.151), it had the smallest *P* value compared with Q12 and Q34 (Supplementary Figure 4B and C). Thus, the cohort was divided into *MAP7*^high^ and *MAP7*^low^ groups according to the median of all samples for further analysis. The Kaplan-Meier method and log-rank test were used to estimate the association between *MAP7* expression and OS/EFS. Fisher’s exact test and Wilcoxon rank-sum test were used to investigate the associations between *MAP7* expression levels and clinical and molecular characteristics for categorical and continuous variables, respectively. Multivariable Cox proportional hazards models were used to study the association between *MAP7* expression levels and OS/EFS in the presence of other known risk factors. Between *MAP7*^high^ and *MAP7*^low^ groups, Student’s *t*-test and multiple hypothesis corrections (False Discovery Rate, FDR) were used to identify differences in gene/microRNA expression and genome-wide promoter methylation profiles. The statistical cutoff values were a fold-change of 1.5 and an adjusted *P*-value of ≤ 0.05. All analyses were performed using the R 3.1.1 software packages.

## Additional Information

**How to cite this article**: Fu, L. *et al*. High expression of *MAP7* predicts adverse prognosis in young patients with cytogenetically normal acute myeloid leukemia. *Sci. Rep.* **6**, 34546; doi: 10.1038/srep34546 (2016).

## Supplementary Material

Supplementary Information

## Figures and Tables

**Table 1:** Patients’ characteristics in the first cohort of 129 CN-AML patients according to *MAP7* expression levels.

Variable	*MAP7*^high^, n = 64	*MAP7*^low^, n = 65	*P*
Median age, y (range)	47.5 (18–59)	45 (16–59)	0.55
FAB subtype, no (%)			
M0	2 (3.1)	0 (0.0)	0.24
M1	21 (32.8)	20 (30.8)	0.85
M2	15 (23.4)	7 (10.8)	0.06
M4	8 (12.5)	13 (20.0)	0.34
M5	14 (21.9)	21 (32.3)	0.24
M6	1 (1.6)	0 (0.0)	0.5
Other	3 (4.7)	4 (6.2)	1
*FLT3*-ITD, no (%)	37 (57.8)	21 (32.3)	0.005
*FLT3*-TKD, no (%)	9 (14.0)	9 (13.8)	1
*NPM1*^*Mut*^*/FLT3*^*WT*^, no (%)	9 (14.0)	10 (15.4)	1
*CEBPA*, mutated, no (%)			
Single	3 (4.7)	1 (1.5)	0.37
Double	2 (3.1)	13 (20.0)	0.005
*IDH1* mutated, no (%)	8 (12.5)	10 (15.4)	0.8
*IDH2* mutated, no (%)	8 (12.5)	4 (6.15)	0.24
High *ERG*, no (%)	42 (65.6)	22 (33.8)	0.0004
High *BAALC*, no (%)	34 (53.1)	30 (46.2)	0.48
High *LEF1*, no (%)	27 (42.1)	37 (57.0)	0.11
High *WT1*, no (%)	50 (78.1)	14 (21.5)	<0.0001
High *DNMT3B*, no (%)	45 (70.3)	19 (29.2)	<0.0001
High *DNMT3A*, no (%)	38 (59.4)	26 (40.0)	0.03
High *MAPKBP1*, no (%)	39 (61.0)	25 (38.5)	0.01
High *ITPR2*, no (%)	40 (62.5)	24 (36.9)	0.005
High *ATP1B1*, no (%)	43 (67.2)	21 (32.3)	<0.001

**FAB**, French-American-British classification; **ITD**, internal tandem duplication; **TKD**, tyrosine kinase domain; **WT**: wild type.

High *ERG, BAALC, LEF1, WT1, DNMT3B, DNMT3A, MAPKP1, ITPR2* and *ATP1B1* expression were defined as an expression level above the median of all samples. *NPM1*^*Mut*^*/FLT3*^*WT*^ was defined as CN-AML patients with a mutation of *NPM1 (NPM1*^*Mut*^) and without *FLT3-*ITD/TKD (*FLT3*^*WT*^).

**Table 2:** Multivariable analysis of OS and EFS in the first cohort of 129 CN-AML patients.

Variables in Final Model by End Point	HR	95% CI	P
OS			
*MAP7* expression	1.7	1.05–2.74	0.03
*NPM1*, mutated *VS* wild type	0.63	0.38–1.05	0.07
*CEBPA*, mutated *VS* wild type	0.57	0.27–1.21	0.14
*IDH1*, mutated *VS* wild type	0.95	0.48–1.85	0.87
*IDH2*, mutated *VS* wild type	0.58	0.25–1.36	0.21
EFS			
*MAP7* expression	1.79	1.13–2.84	0.01
*NPM1,* mutated *VS* wild type	0.52	0.31–0.86	0.01
*CEBPA,* mutated *VS* wild type	0.69	0.35–1.37	0.29
*FLT3*-ITD, presented *VS* others	1.6	1.0–2.55	0.04
*IDH1*, mutated *VS* wild type	1.31	0.71–2.4	0.39
*IDH2*, mutated *VS* wild type	0.65	0.28–1.53	0.32

**HR**, hazard ratio; **CI**, confidence interval.
