# Supplementary material for: High expression of MAP7 predicts adverse prognosis in young patients with cytogenetically normal acute myeloid leukemia
Source: Sci Rep. 2016 Sep 30;6:34546. doi: 10.1038/srep34546 (PMC5043276; doi:10.1038/srep34546)
Supplement: Supplementary Information [file srep34546-s1.pdf]

High expression of *MAP7* predicts adverse prognosis in young patients with cytogenetically normal acute myeloid leukemia

Lin Fu[1, 2, 3*], Huaping Fu[4,*], Lei Zhou[5, *], Keman Xu[6], Yifan Pang[7], Kai Hu[1], Jing Wang[1], Lei Tian[1], Yuanyuan Liu[1], Jijun Wang[1], Hongmei Jing[1], Wenrong Huang[5], Xiaoyan Ke[1], Jinlong Shi[2]

[1]Department of Hematology and Lymphoma Research Center, Peking University, Third Hospital, Beijing, 100191, China

[2]Department of Biomedical Engineering, Chinese PLA General Hospital, Beijing, 100853, China

[3]Blood Diseases Institute, Xuzhou Medical University, Xuzhou, 221002, China

[4]Department of nuclear medicine, Chinese PLA General Hospital, Beijing, 100853, China

[5]Department of Hematology, Chinese PLA General Hospital, Beijing, 100853, China

[6]College of medical laboratory science and technology, Harbin Medical University, Daqing, 163319, China

[7]Department of Medicine, William Beaumont Hospital, Royal Oak, MI, 48073, USA

[*]These authors contributed equally to this work.

Correspondence and requests for materials should be addressed to:

J.L.S. (E-mail: jinlong_301@163.com) or X.Y.K. (E-mail: xiaoyank@yahoo.com) or W.R.H (E-mail: huangwr301@163.com)

Table S1. Characteristics of the 88 CN-AML patients in the second independent cohort segregated based on *MAP7* expression level

**Variable**	***MAP7*$^{\mathbf{high}}$, n=44**	***MAP7*$^{\mathbf{low}}$,n=44**	***P***
Median age. y (range)	46 (19-59)	47(17-59)	0.67
FAB subtype, no (%)			
M0	0 (0.0)	3 (6.8)	0.24
M1	13 (29.5)	7 (15.9)	0.21
M2	11 (25.0)	9 (20.5)	0.8
M4	16 (36.4)	12 (27.2)	0.49
M5	3 (6.8)	10 (22.7)	0.033
M6	1 (2.3)	3 (6.8)	0.62
High *ERG*, no (%)	31 (70.5)	13 (29.5)	<0.001
High *BAALC*, no (%)	21 (47.8)	23 (52.3)	0.83
High *LEF1*, no (%)	14 (31.8)	30 (68.2)	0.001
High *WT1*, no (%)	34 (77.3)	10 (22.7)	<0.001
High *DNMT3B*, no (%)	33 (75.0)	11 (25.0)	<0.001
High *DNMT3A*, no (%)	28 (63.6)	16 (36.4)	0.02
High *MAPKBP1*, no (%)	31 (70.5)	13 (29.5)	<0.001
High *ITPR2*, no (%)	37 (84.1)	7 (15.9)	<0.001
High *ATP1B1*, no (%)	33 (75.0)	11 (25.0)	<0.001

FAB, French-American-British classification.

High *ERG*, *BAALC*, *LEF1*, *WT1*, *DNMT3B*, *DNMT3A*, *MAPKP1*, *ITPR2* and *ATP1B1* expression were defined as an expression level above the median of all samples, respectively.

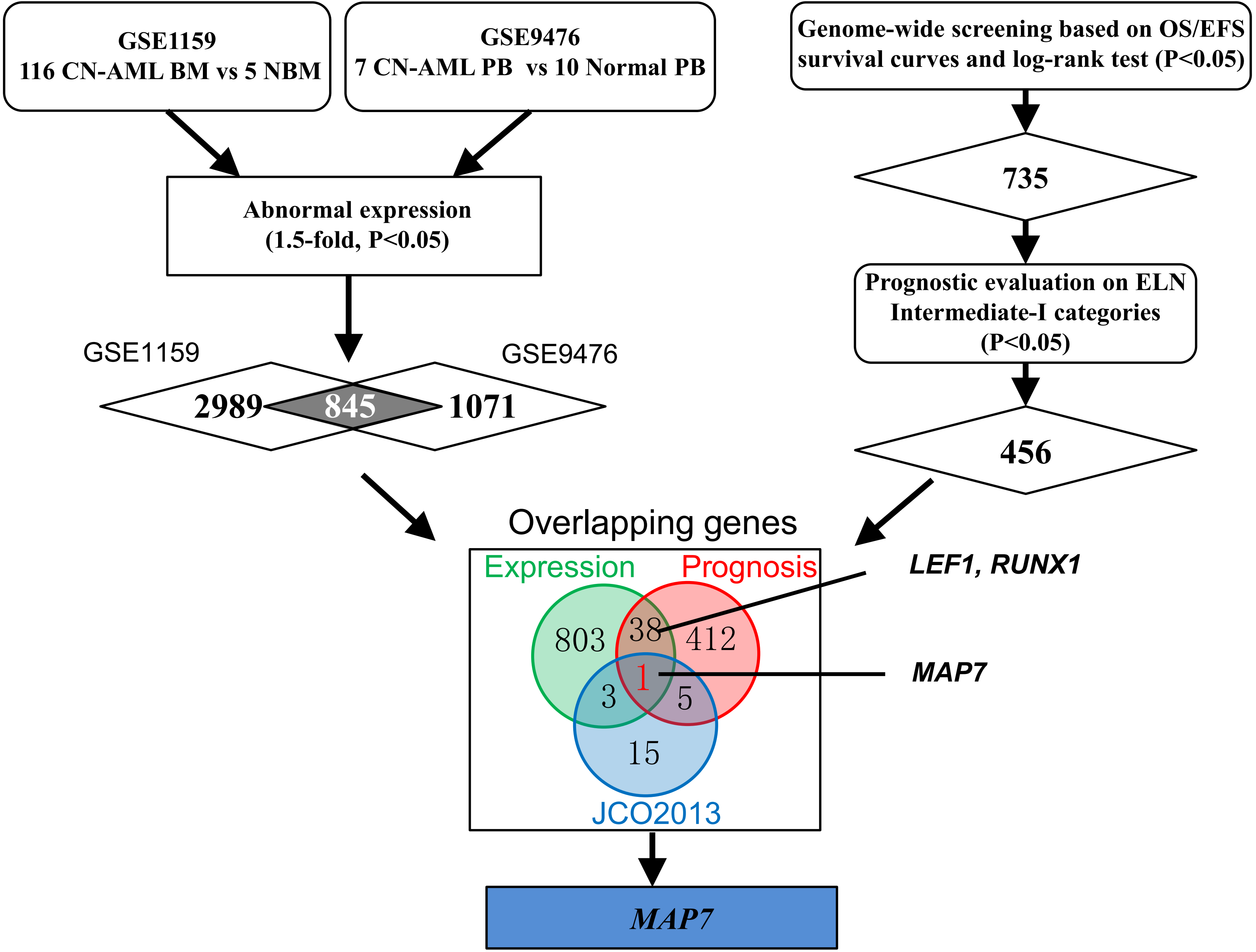

Supplementary Figure 1. A schematic diagram of the systematic strategies for uncovering the prognostic significance of *MAP7* in CN-AML patients.

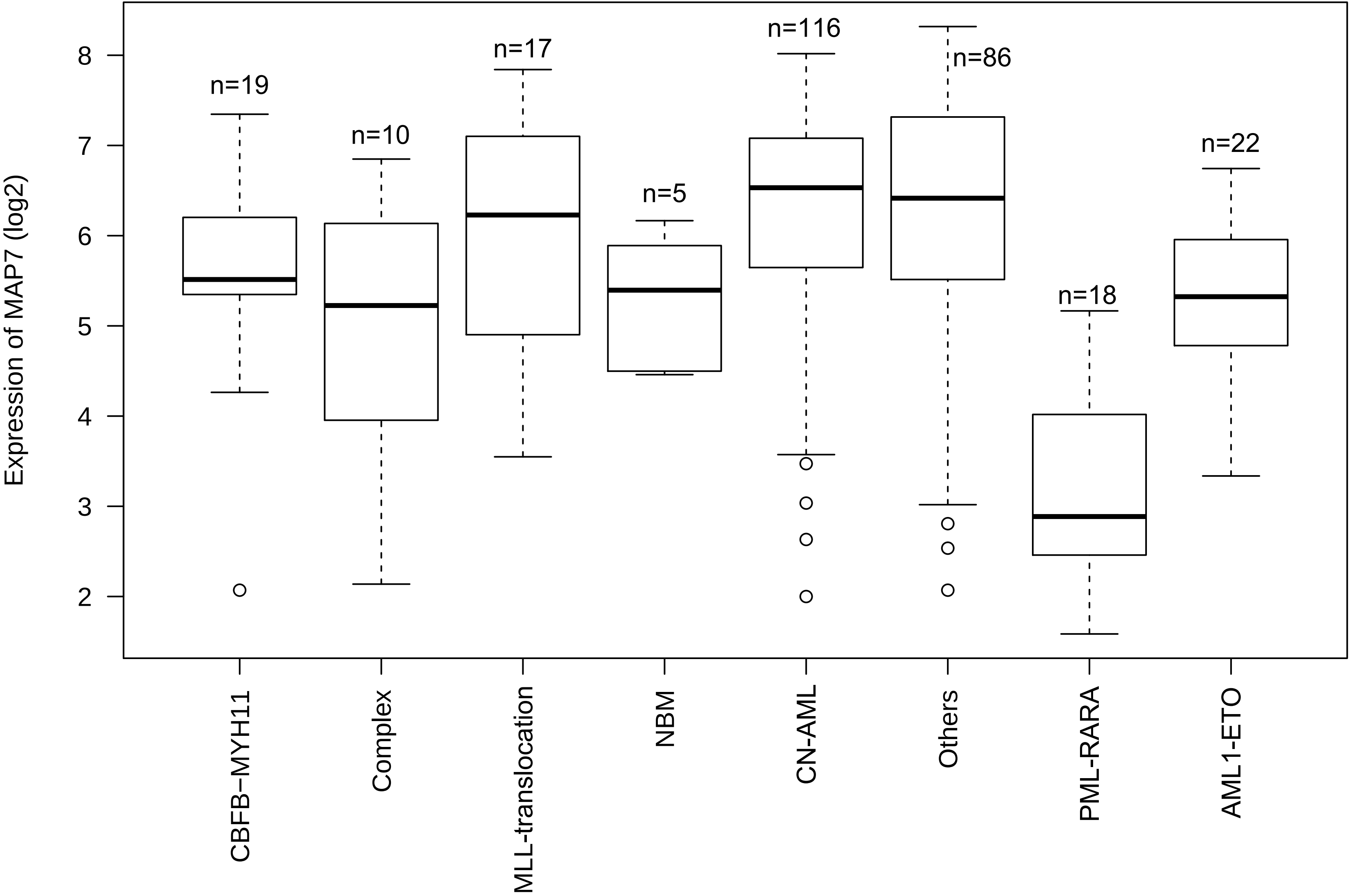

Supplementary Figure 2. *MAP7* levels in various different subgroups of AML patients.

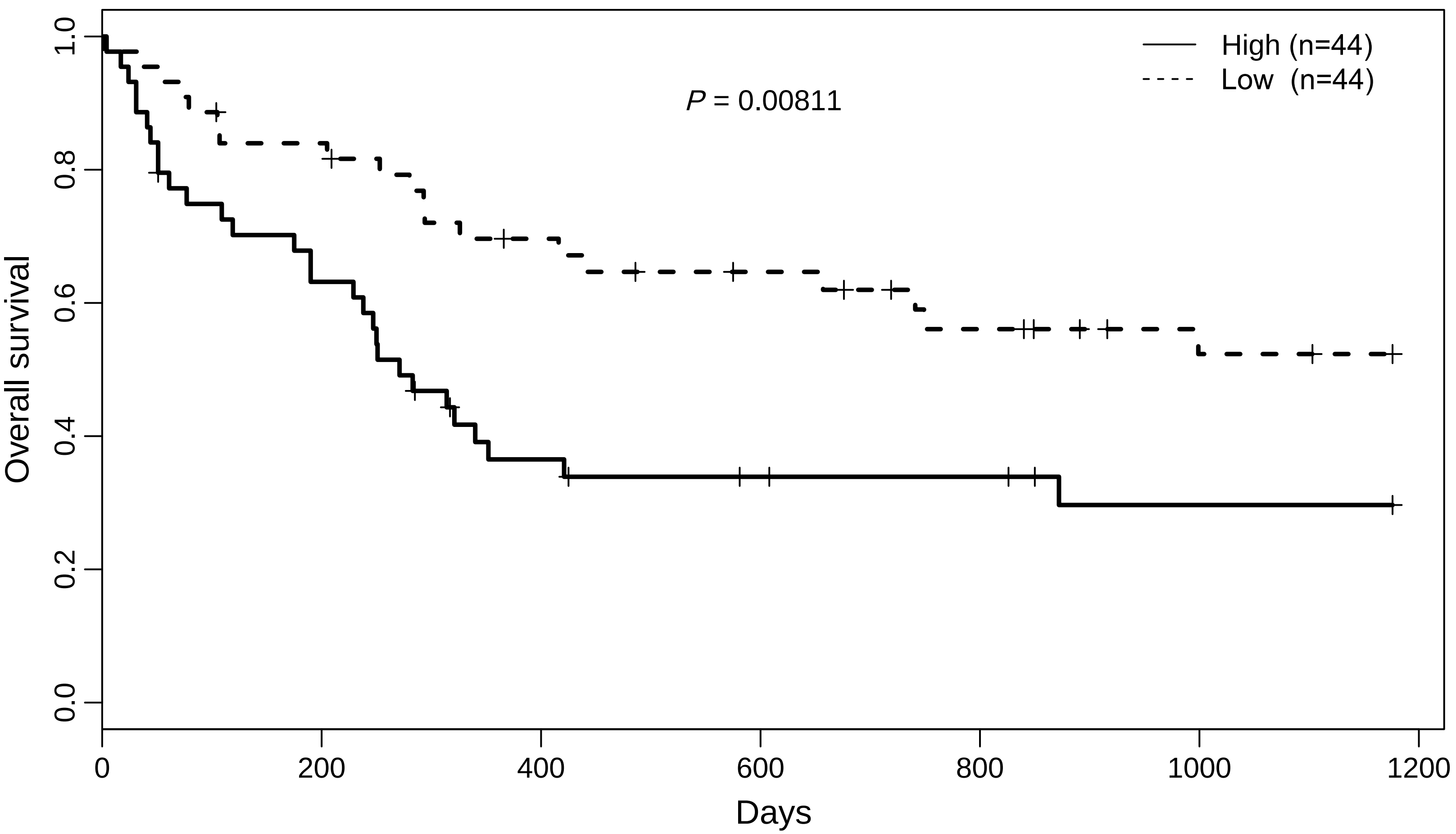

Supplementary Figure 3. OS of CN-AML patients in the second independent

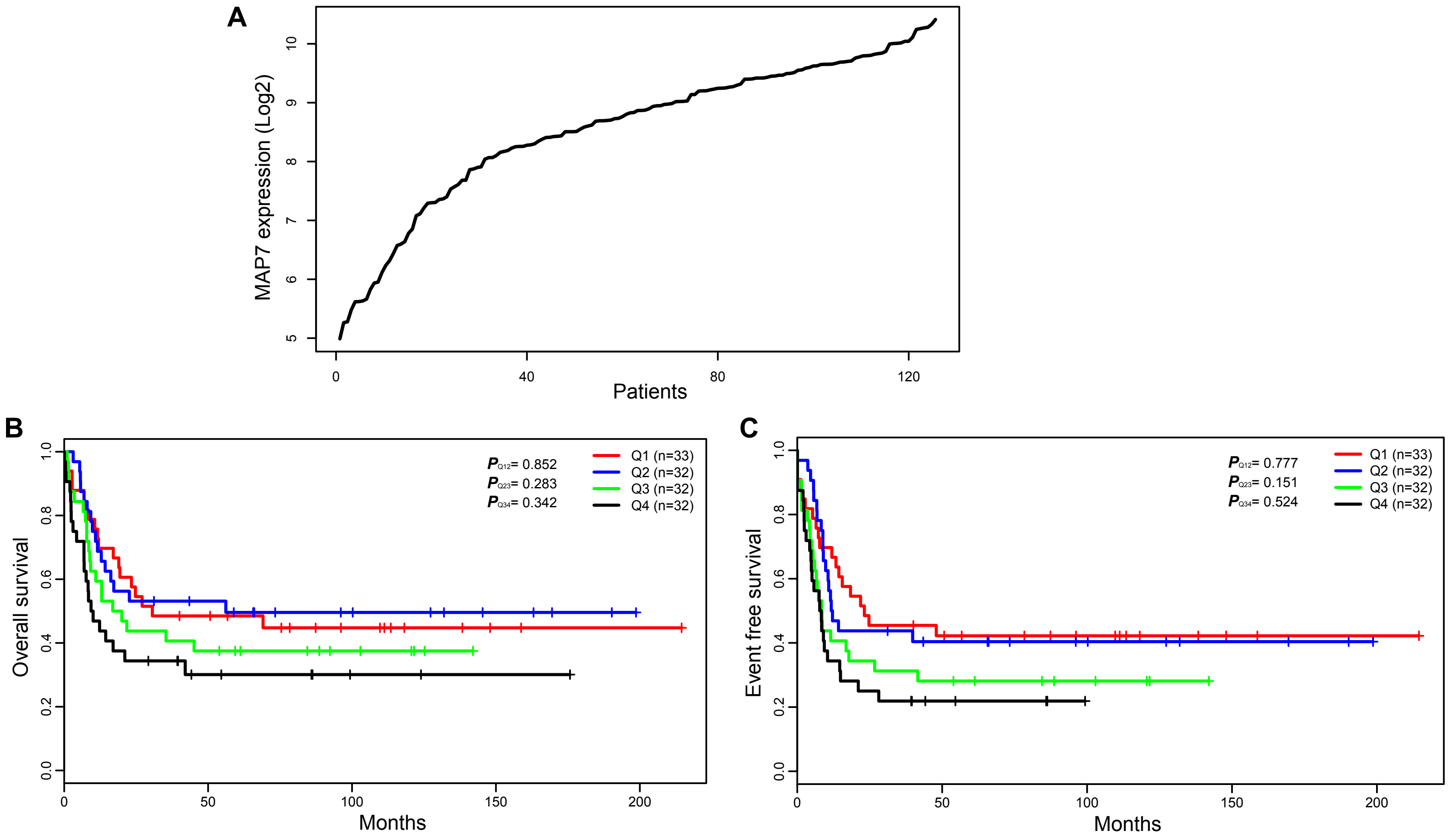

Supplementary Figure 4. Median value of *MAP7* expression as the cut-off. (A) *MAP7* expression is normally distributed. (B) OS and (C) EFS AML patients were subdivided into four quartiles based on the quartile of *MAP7* expression.
